# Do people with arthritis differ from healthy controls in their internal comparison standards for self-reports of health, fatigue, and pain?

**DOI:** 10.1186/s41687-019-0108-3

**Published:** 2019-03-27

**Authors:** Doerte U. Junghaenel, Stefan Schneider, Arthur A. Stone

**Affiliations:** 1 USC Dornsife Center for Self-Report Science, & Center for Economic and Social Research, University of Southern California, Verna & Peter Dauterive Hall, 635 Downey Way, Los Angeles, CA 90089-3332 USA; 2 Department of Psychology, University of Southern California, Los Angeles, CA USA

## Abstract

**Objective:**

Patient-reported outcomes are central for the assessment and treatment of people with chronic disease. The primary aim of this study was to determine if people with arthritis differed from healthy individuals in their use of internal comparison standards when answering questions about their health and symptomatology. A secondary aim was to determine if average levels of the patient-reported outcomes were associated with the use of internal comparison standards, regardless of whether a participant had arthritis or no chronic medical condition.

**Methods:**

People with a self-report diagnosis of any type of arthritis (*n* = 588) and healthy controls (*n* = 567) were recruited from an Internet panel and were randomly assigned to rate two of three outcomes: health, fatigue, and pain. After completing their rating, participants were presented with internal comparison standards and indicated which ones, if any, they used for the ratings they provided. The internal comparison standards were: *Interpersonal* (comparisons with other people); *Historical* (comparisons with the past); *Imaginary* comparisons (comparisons with a hypothetical scenario); or that none of the three were used.

**Results:**

After controlling for group differences in demographic characteristics and outcome levels by including them in the analyses as covariates, people with arthritis were more likely to make *Historical* comparisons than healthy controls when rating their health. No other group differences in the use of internal comparison standards were found. We further found that the use of internal comparison standards was associated with health and symptom levels, regardless of whether a participant had arthritis or no medical condition. Poorer self-rated health, greater fatigue, and higher pain were associated with a greater likelihood of making a *Historical* comparison. Furthermore, poorer self-rated health was associated with a greater likelihood of making an *Interpersonal* comparison, and higher fatigue and pain with a greater likelihood of making an *Imaginary* comparison.

**Conclusion:**

People with arthritis differed in their use of *Historical* comparison standards compared to those with no chronic medical condition for health ratings. In addition, poorer health and more severe symptomatology were associated with the use of internal comparison standards in both groups of participants, people with arthritis and healthy controls.

## Introduction

Patient-reported outcomes of health and symptomatology are key ingredients in the assessment and treatment of chronic pain [1, 2] and are central to the evaluation of clinical outcomes [2–4]. Given the importance of patient self-report, it seems prudent to increase our understanding about how patients form their judgments about health-related experiences [5, 6]. Patients with chronic medical conditions face a number of significant challenges that might change the way they view themselves. [6–10]. For example, the literature on response shift posits that the meaning of patients’ self-evaluations might change as a result of modifications to their values, their experiences, their definitions of what health, quality of life, and day-to-day functioning mean to them, as well as changes to their internal comparison standards when rating their health and well-being [6, 11, 12]. Understanding what these internal comparison standards are and when they are used can facilitate our interpretation of patient-reported outcomes and of group-differences in health-related experiences [5, 6, 12]. To illustrate this point, studies have found that asking individuals to rate their health “compared to someone their age” is associated with less pronounced age differences than health ratings for items without a stated comparison standard [13, 14].

To date, we know little about the internal comparison standards that patients with chronic medical conditions might apply in their self-report ratings. In this paper, we focused on people with arthritis. Most self-report questions do not include a comparison standard in the question wording leaving the choice (if any) up to the person [15]. If patients systematically differ from healthy people in their internal comparison standards for outcome measures, the comparability of responses from medical and healthy populations could be compromised.

The study had two aims. (i) The primary aim was to test whether or not internal comparison standards for self-rated health, fatigue, and pain reported by people with arthritis differed from those reported by healthy individuals. We expected that people with arthritis would prefer different internal comparison standards compared to people without a chronic medical condition. We expanded on prior research by studying three distinct patient-reported outcomes in arthritis and by examining differences between patients and healthy individuals in the use of different types of internal comparison standards. (ii) The secondary aim was to test whether or not the level of self-reported health, fatigue, and pain was associated with differences in the use of internal comparison standards, regardless of whether a person had arthritis or had no medical conditions. We hypothesized that poorer health and higher symptom levels would be associated with different internal comparison standards than better health and lower symptomatology. While the first aim allowed us to examine whether a diagnosis of a chronic medical condition was associated with internal comparison standards that are different from those used by healthy individuals, the second aim examined whether a person’s level of health and symptomatology was associated with internal comparison standards, regardless of whether the person had or had not been diagnosed with a chronic medical condition.

## Methods

### Participants and procedure

This study reports on secondary analysis of an existing dataset [16]. Participants were recruited from a U.S. national Internet panel of about one million households, hosted by Survey Sampling International (SSI). The opt-in panel includes people who volunteered to periodically take part in paid Internet surveys. Participants’ diagnosis of arthritis was collected via self-report.

Participants completed the questionnaire online and were randomly assigned to report on two of the three patient-reported outcomes (i.e., health, fatigue, and pain) examined here. Participants first provided their self-report rating for one of the two selected outcomes. Next, they were presented with a list of internal comparison standards. These were derived from prior extensive qualitative work in our team [17]. Participants were asked to indicate which of these, if any, they were thinking about for their rating: *Interpersonal comparisons* (“I compared myself with another person or other people”), *Historical comparisons* (“I made a comparison with how I was some time ago”), *Imaginary comparisons* (“I thought about how I would feel if something about me or my life were different”). They also had an option to say that they were not thinking of any of the internal comparison standards. Participants could check all options that applied. These steps were repeated for both outcome variables. The online branching system also administered other survey questions not reported in the present study. Information about the internal comparison standard checklist is available from the authors.

### Measures

To characterize participants’ medical history, they were asked to state for each of 16 chronic health conditions, “Has a doctor, nurse, or other health professional ever told you that you had the following?” [18]. The medical condition checklist was taken from the Patient-Reported Outcomes Measurement Information System (PROMIS®) wave 1 instrument testing with general population and clinical samples [1]. It included a question about “arthritis” (no type specified) along with other diagnoses, such as cardiac, neurological, psychiatric, and pulmonary medical conditions. Participants who endorsed that they were diagnosed with arthritis on the medical condition checklist defined the arthritis group. The comparison group was comprised of healthy controls. To be included in this group, only those participants who did not endorse any of the 16 medical conditions were considered for the analyses.

The self-rated health, fatigue, and pain items did not have a specific comparison standard in the item wording. For subjective health, the global health item from the SF-36v2 [19, 20] was used (“In general, would you say your health is …” ). The fatigue question was from the Brief Fatigue Inventory [21] (“Please rate your fatigue (weariness, tiredness) by circling the one number that best describes your usual level of fatigue”). The pain question was from the Brief Pain Inventory [22] (“Please rate your pain by circling the number that best describes your pain on the average”).

### Data analysis

Data analyses were conducted in STATA 14. Whether or not the use of internal comparison standards differed between the groups was examined in a series of binary logistic regressions, conducted separately for each of the three internal comparison standards (i.e., *Historical, Interpersonal, and Imaginary* comparison) and patient-reported outcomes. In each regression, the presence or absence of a given internal comparison standard was the dependent variable and group (i.e., arthritis vs. healthy control) was the independent variable. Due to significant group differences on demographic characteristics, health, and symptom level, the analyses controlled for participants’ age (linear), gender, race, socioeconomic status, ethnicity, and level of the health-related outcome (i.e., self-report rating for health, fatigue, and pain) by introducing them as covariates into the analyses. Participant ratings of health, fatigue, and pain were standardized (z-scores) in the analyses to facilitate interpretation. In supplementary analyses, we further explored evidence for interaction effects between group (arthritis vs. health control) and each of the demographic variables in relation to respondents’ comparison standards. These moderator analyses address the potential heterogeneity of group differences in comparison standards by asking whether these group differences depend on demographic individual difference variables.

## Results

### Participant sample

Table 1 presents the demographic characteristics for patients with arthritis (*n* = 588) and healthy controls (*n* = 567). People with arthritis were older, significantly more likely to be female and Caucasian, had lower socioeconomic status, and were significantly less likely to be Hispanic. People with arthritis had significantly lower self-rated health, higher fatigue, and higher pain ratings compared to healthy controls. There were only 7.4% of instances in which individuals selected more than one standard of comparison from the response options list.

**Table 1:** Demographic characteristics and self-report ratings of people with arthritis and healthy controls

	People with Arthritis (*n* = 588)	Healthy Controls (*n* = 567)
Gender, female %*	62	48
Age, mean (SD)*	61.8 (11.6)	45.8 (15.6)
Marital Status, married/living as married %	55	59
Education, %		
Up to High School	23	18
College or College Graduate	61	67
Graduate Degree	16	15
Race, Caucasian %*	75	62
Ethnicity, Hispanic %*	9	19
Socioeconomic Status, higher %*	62	74
Global health rating (1–5 scale), mean (SD)	2.89 (1.00)	3.91 (0.87)
Fatigue rating (0–10 scale), mean (SD)	4.75 (2.46)	3.40 (2.56)
Pain rating (0–10 scale), mean (SD)	4.78 (2.51)	2.50 (2.74)

*Note:* *Significant difference between the two groups: *p ≤ .001*

### Differences in internal comparison standards between people with arthritis and healthy controls

Our primary aim was to test whether people with arthritis differ in their use of internal comparison standards from healthy people. Table 2 presents the frequency of the use of internal comparison standards by study group and outcome. After controlling for differences in demographic characteristics and outcome levels, people with arthritis were more likely to make *Historical* comparisons than healthy controls when rating their health (OR = 3.56, SE = 0.88, *p* < .001). No other significant group differences in the use of internal comparison standards were found (see Table 3). We did not find any significant interactions between group and any of the demographic variables in relation to the use of internal comparison standards (all *p*s > .20).

**Table 2:** Use of *Historical, Interpersonal,* and *Imaginary c*omparisons in people with arthritis and healthy controls

	Historical Comparison	Interpersonal Comparison	Imaginary Comparison	None of the FoRs
Health				
Arthritis	46%	21%	12%	33%
Healthy Control	25%	25%	15%	43%
Fatigue				
Arthritis	42%	6%	12%	43%
Healthy Control	32%	12%	14%	49%
Pain				
Arthritis	42%	8%	15%	43%
Healthy Control	29%	14%	16%	49%

*Note:* Percentages are not controlled for demographic and outcome level differences

**Table 3:** Multiple logistic regressions predicting *Historical, Interpersonal*, and *Imaginary* comparison standards for ratings of health, fatigue, and pain

	Historical		Interpersonal		Imaginary	
	*OR*	95% CI	*OR*	95% CI	*OR*	95% CI
Health						
Group (arthritis vs healthy controls)	3.56***	2.20–5.78	1.36	0.82–2.27	0.66	0.35–1.24
Health rating (z-scores)	0.81*	0.66–1.00	1.33*	1.05–1.69	0.77	0.58–1.02
Female gender	0.59**	0.41–0.85	0.73	0.49–1.09	1.02	0.63–1.66
Age (linear effect per 10-years)	0.77***	0.67–0.89	0.84*	0.73–0.98	0.91	0.76–1.10
White race	1.16	0.70–1.92	1.77	0.96–3.25	1.21	0.62–2.33
Hispanic	1.63	0.88–3.04	0.99	0.46–2.10	0.95	0.41–2.21
High socioeconomic status	0.97	0.66–1.45	1.91*	1.17–3.12	0.67	0.40–1.11
Fatigue						
Group (arthritis vs healthy controls)	1.29	0.83–1.99	1.37	0.62–3.02	0.88	0.48–1.65
Fatigue rating (z-scores)	1.53***	1.26–1.85	1.12	0.82–1.53	1.45**	1.11–1.90
Female gender	0.79	0.55–1.14	0.52*	0.28–0.97	0.84	0.50–1.41
Age (linear effect per 10-years)	1.04	0.90–1.19	0.56***	0.44–0.72	0.84	0.69–1.01
White race	1.58	0.96–2.58	1.02	0.46–2.26	1.41	0.71–2.81
Hispanic	1.39	0.70–2.73	1.25	0.47–3.29	1.02	0.39–2.62
High socioeconomic status	1.51*	1.02–2.21	1.21	0.60–2.41	0.70	0.42–1.18
Pain						
Group (arthritis vs healthy controls)	1.44	0.91–2.29	0.91	0.44–1.88	0.75	0.41–1.38
Pain rating (z-scores)	1.33**	1.08–1.63	1.30	0.96–1.75	1.67***	1.29–2.18
Female gender	0.88	0.61–1.27	0.56	0.32–1.00	0.91	0.56–1.46
Age (linear effect per 10-years)	0.99	0.86–1.14	0.68**	0.54–0.84	0.91	0.75–1.09
White race	1.15	0.71–1.86	2.15	0.86–5.36	0.85	0.46–1.57
Hispanic	0.69	0.35–1.37	1.91	0.66–5.52	1.04	0.46–2.35
High socioeconomic status	1.51*	1.02–2.23	1.00	0.55–1.83	0.87	0.53–1.42

*Note*: OR = odds ratio; CI = confidence interval. *p < .05; **p < .01; ***p < .001

### Does the level of self-rated health, fatigue and pain predict the use of internal comparison standards regardless of group assignment?

The secondary aim was to test whether the level of the self-report rating was associated with the use of internal comparison standards regardless of whether a person was diagnosed with arthritis or was a healthy control. We found that poorer self-rated health (OR = 0.81 SE = 0.09, *p* = .048), greater fatigue (OR = 1.53, SE = 0.15, *p* < .001), and higher pain severity ratings (OR = 1.33, SE = 0.14, *p* = .006) were each associated with a greater likelihood to make a *Historical* comparison than to not make this comparison after controlling for demographic differences and group assignment. Poorer self-rated health was also associated with a greater likelihood of making an *Interpersonal* comparison than to not make this comparison (OR = 1.33, SE = 0.16, *p* = .019). Furthermore, we found that higher fatigue (OR = 1.45, SE = 0.20, *p* = .006) and higher pain severity ratings (OR = 1.67, SE = 0.22, *p* < .001) were associated with a greater likelihood of making an *Imaginary* comparison than to not make this comparison (see Table 3).

## Discussion

Patients’ perceptions of their health and symptomatology are at the core of assessment and treatment of chronic pain [1, 2, 23]. Understanding how patients formulate their responses can provide critical guidance for interpretations of the accuracy and validity of self-report outcomes [6, 11, 12]. The primary goal of the present study was to compare internal comparison standards for common patient-reported outcomes between people with arthritis and healthy individuals.

For *Historical* comparisons, we found that people with arthritis were significantly more likely to make these comparisons for self-rated health than healthy individuals. This result is in line with prior research on health-related quality of life in Paget’s disease [24]. Fayers et al. (2007) found that the majority of patients considered how they felt in the past and before their medical condition. It is possible that people with arthritis in the present study thought about a time before their diagnosis or onset of their medical condition, an internal comparison standard that is likely specific and very salient to them when rating their overall health. On the other hand, healthy individuals may not have had such a salient reference that drew them towards making comparisons with the past. A similar pattern of results was found for the use of *Historical* comparisons for pain and fatigue, although the group differences did not reach statistical significance.

We found no group differences in the use of *Interpersonal* comparisons. This was somewhat surprising in that prior work on social comparisons has found socio-comparative reference points to be common among patients with chronic illness [7]. One point might partially explain these results. Prior studies have frequently focused on examinations within patient groups rather than comparisons with healthy controls. Our results suggest that the tendency to draw on one’s social network as a point of reference also extends to healthy samples. In addition, no group differences were found for *Imaginary* comparisons and the prevalence of *Interpersonal* and *Imaginary* comparisons was relatively low compared to *Historical* comparisons. Participants completed outcome measures in an internet survey and many likely completed it in their home environment. Had we sampled self-report ratings in a different environmental context, such as a clinic waiting room or in a group setting, the salience of other people might have been heightened particularly for patients.

Our secondary aim was to examine whether levels of health and symptomatology were associated with differences in the use of internal comparison standards, regardless of whether a participant had arthritis or no medical condition. We found that poorer self-rated health, greater fatigue, and higher pain severity were all significantly associated with a greater likelihood of reporting a *Historical* comparison. Furthermore, poorer self-rated health was associated with a greater likelihood of making an *Interpersonal* comparison, and higher fatigue and pain with a greater likelihood of making an *Imaginary* comparison. It should be noted that the Odds Ratios from these analyses indicate small (but possibly not trivial) effects following common effect size conventions, which may be just at the cusp of differences that may be deemed clinically meaningful [25]. The results suggest that a diagnosis of a chronic medical condition in and of itself might not be associated with the use of internal comparison standards, but rather elevated levels of symptomatology and poorer health. Future research with more clinical and non-clinical samples could examine whether this hypotheses generated from our data can be confirmed. Similarly, prospective cohort studies could shed light on whether the use of internal comparison standards might change as a function of differing symptom levels in participants over time.

The present study has limitations. Participants were recruited through Internet sampling, which creates the possibility for potential participant selection bias. In addition, we did not collect data on the demographic and medical characteristics of those people who declined participation in the study. Patients’ diagnosis of arthritis was based on self-report. Future research needs to examine whether our results replicate in clinical samples. Other pertinent medical information, such as time since diagnosis or arthritis disease severity, was not collected. This may matter because prior research suggests that patients’ internal comparison standards might change as a function of time since diagnosis [7]. The selection of the three internal comparison standards was based on prior qualitative work in our team [17]. It is, however, possible that an individual’s range of internal comparison standards might be broader than the ones presented in the paper and future research could examine whether there are other salient comparison standards in patients with medical conditions. Furthermore, it is important to note that we applied a nomothetic approach to the study of internal comparisons standards and our results should not be understood as to mean that all people with arthritis make the same type of comparison. Future research would benefit from augmenting the presented approach by including measures of standards of comparison appraisal that are suited to consider idiographic differences. Finally, due to the cross-sectional nature of our study design, a causal relationship between the use of internal comparison standards and health ratings cannot be established.

We would like to outline suggestions for future research. Future research would benefit from examining internal comparison standards for other patient-reported outcomes, such as positive and negative affect and patients’ physical functioning in other medical conditions. In addition, prior research has suggested that the use of internal comparison standards might fluctuate across time [24]. Repeated assessments over the span of weeks or months might shed light on the temporal stability and change in patients’ natural use of internal comparison standards.

In sum, we found only one significant difference in the use of internal comparison standards between people with arthritis and healthy controls: People with arthritis used *Historical* comparisons more often than those without the disease for health ratings. Regardless of group assignment, we found that poorer health and higher symptom levels are likely to evoke internal comparison standards, even if no such comparisons are intended or made explicit in the instructions or wording of an outcome measure.

## Abbreviations

PROMIS®: Patient-Reported Outcomes Measurement Information System
